# Network pharmacology and molecular docking approach to elucidate the mechanisms of safflower, phellodendron, scutellaria baicalensis, coptis, and gardenia in hand–foot syndrome

**DOI:** 10.3389/fmed.2024.1454776

**Published:** 2024-09-16

**Authors:** Pengxing Li, Lizhu Chen, Jianhui Liu

**Affiliations:** ^1^Department of Gastrointestinal Surgery, Shaowu Municiple Hospital of Fujian Province, Nanping, China; ^2^Department of Medical Oncology, Clinical Oncology School of Fujian Medical University, Fujian Cancer Hospital, Fuzhou, China; ^3^Department of Traditional Chinese Medicine, Shaowu Municiple Hospital of Fujian Province, Nanping, China

**Keywords:** hand-foot syndrome, molecular mechanism, network pharmacology, traditional Chinese medicine, molecular docking

## Abstract

**Background:**

Safflower, phellodendron, scutellaria baicalensis, coptis, and gardenia (SPSCG) are medicinal plants with a wide range of anti-inflammatory and antioxidant effects. However, the related mechanism of SPSCG against hand-foot syndrome (HFS) has yet to be revealed.

**Objective:**

To investigate the mechanisms of SPSCG in the treatment of HFS using the Network Pharmacology.

**Methods:**

Active ingredients and targets of SPSCG for HFS were screened by the Chinese Medicine Systems Pharmacology (TCMSP) and Swiss Target Prediction databases. Potential therapeutic targets were collected from the GeneCards and OMIM databases. Subsequently, protein–protein interactions (PPI), Gene Ontology (GO) annotations, and pathways from the Kyoto Encyclopedia of Genes and Genomes (KEGG) were performed to investigate the potential mechanism of the SPSCG in HFS. Then, molecular docking and molecular dynamics simulations were performed to predict the binding interactions between the active compound and the core target. Finally, vitro experiments were used to verify the repair effect of key ingredients of SPSCG on cell damage caused by 5-Fluorouracil.

**Results:**

Quercetin, kaempferol, β-sitosterol, and stigmasterol were identified as the major active components of SPSCG. GO analysis showed a total of 1,127 biological processes, 42 terms cellular components, and 57 molecular functions. KEGG analysis showed that the MAPK, TNF, and IL-17 signaling pathways were significantly enriched. The PPI analysis discovered that EGFR, CASP3, AKT1, CCND1, and CTNNB1 shared the highest centrality among all target genes. The experimental results confirmed that these SPSCG active ingredients could treat HFS by reducing inflammation reaction and promoting cell damage repair.

**Conclusion:**

SPSCG may alleviate HFS by exerting antioxidative effects and suppressing inflammatory responses.

## Introduction

Hand–foot syndrome (HFS) is a prevalent cutaneous toxic reaction to chemotherapy in various malignancies (1), which was initially identified and documented by Dr. Zuehlke in 1974 (2). HFS typically occurs in the palms of the hands and on the soles of the feet, with symptoms such as numbness, tingling, ulcers, skin peeling, or even erosion, greatly affecting patients’ life quality (3). The clinical manifestations of HFS vary depending on the drug, treatment protocol, dosage, plasma concentration, and treatment duration. The incidence of HFS ranges from 6 to 64% (4). HFS is thought to be caused by an inflammatory response (5) associated with the proinflammatory receptor enzyme COX-2 (cyclooxygenase 2) and the accumulation and metabolism of antimetabolites (6). Therefore, in Western Medicine, the management of HFS involves reducing the dose of chemotherapy and increasing the interval between cycles, or discontinuing the treatment. Drug therapy for HFS primarily includes the use of oral celecoxib (7) or glucocorticoids (8). However, long-term use of such drugs can have anti-inflammatory-related and hormone-related negative effects. Additionally, any disruption or reduction in anti-cancer treatment can affect treatment outcomes. At present, effective treatment strategies for HFS are lacking. However, plant products hold great promise in the treatment of inflammatory and allergic reactions, with fewer side effects (9). Consequently, plant-derived compounds or plant extracts can be used to develop novel anti-inflammatory drugs for treating HFS.

Safflower, phellodendron, scutellaria baicalensis, coptis, and gardenia (SPSCG) are medicinal plants with a wide range of anti-inflammatory and antioxidant effects. According to the meta-analysis, safflower could alleviate the symptoms of fluorouracil-induced HFS (10). However, the mechanism is uninvestigated. Compound phellodendron also could suppress inflammatory responses by regulating the TLR4/nuclear factor κ-B (NF-κB)/p38 mitogen-activated protein kinase (MAPK) signaling pathway (11). Recent studies demonstrated that scutellaria baicalensis, a perennial herb belonging to the Lamiaceae family, had anti-inflammatory (12–14) and antioxidant activities (15, 16). Coptis exhibited various pharmacological activities, including antibacterial, antiviral, anticancer, and antioxidant process (17). Gardenia (Rubiaceae) is widely used in both TCM and culinary. According to TCM principles, gardenia could access the heart, lung, and tri-jiao meridians, thus reducing pathological internal heat and promoting blood cooling (18). Geniposide has been identified as the primary active constituent of the gardenia (19), which, exerts anti-inflammatory effects by regulating the MAPK/NF-κB and phosphatidyl inositol 3 kinase–protein kinase B (PI3K–Akt) signaling pathways (20). In addition, it induced the IL-10, suppressing the expression of proinflammatory factors and facilitating skin wound healing (21). Hence, it holds some merits in investigating the role of SPSCG in HFS for its anti-inflammatory and antioxidant effects.

Network Pharmacology integrates bioinformatics, multidirectional pharmacology, computer science, and other interdisciplinary fields (22). It can be used to elucidate the potential mechanisms of drug action from various perspectives, reflecting the comprehensive and systematic nature of drug action. Investigating the principles of TCM and syndrome differentiation in TCM is challenging. However, Network Pharmacology is a promising approach to overcoming this challenge and, therefore, has received more attention in research on TCM (23).

In this study, Network Pharmacology was used to identify active components of SPSCG, including quercetin, β-sitosterol, stigmasterol, and kaempferol. We also intersected SPSCG target genes with HFS-related genes to determine more effective drugs for treating HFS. The findings may provide a valuable reference for future studies.

## Materials and methods

### Active components and targets of SPSCG

The active components and targets of SPSCG were identified using the Traditional Chinese Medicine Systems Pharmacology (TCMSP) database and analysis platform. ADMET lab 2.0^1^ was used for the insilico Drug metabolism study. The most commonly used screening parameter for web-based pharmacological analysis was oral bioavailability (OB), and drug-likeness (DL). In this study, the criteria of OB ≥ 30% and drug-likeness ≥0.18 were applied. Active components meeting these criteria were selected, and the corresponding targets were obtained using Mol ID. Gene names were normalized using the UniProt database.^2^

### Identification of HFS-related targets

The keyword “hand-foot syndrome” was applied in the DisGeNET,^3^ OMIM,^4^ and GeneCards^5^ databases to screen, de-duplicate, and integrate HFS-related targets. VENNY2.1.0 was used to intersect the targets of SPSCG and HFS-related targets to obtain shared core targets.

### Construction of the SPSCG-active component-target network

The SPSCG and its corresponding active components and targeted genes were imported into Excel to generate network and attribute files. Then, the Cytoscape (version 3.7.2) software was used to visualize the network, and the built-in tool Network Analyzer was used to assess the topological parameters of the network. Core active components and target genes were identified based on the node degree value (degree).

### Construction of the protein–protein interaction network

To visualize the interaction between active components of SPSCG and HFS-related targets, the overlapping targets were imported into the STRING (version 11.0) database^6^ to establish a protein–protein interaction (PPI) network. The biological species was defined as “*Homo sapiens*,” and the minimum interaction threshold was defined as “medium confidence” (>0.4). TSV files were acquired, and the PPI network was visualized using the Cytoscape (version 3.7.2) software. The Network Analyzer was used to assess the topological parameters of the PPI network, and key targets were identified based on the degree value.

### Enrichment analysis of targets of SPSCG in HFS

The targets of SPSCG related to HFS were imported into the Metascape platform^7^ for the GO and KEGG analysis. Significantly enriched biological processes and signaling pathways were identified based on *p*-values of <0.01. The micro-shengxin online platform was used to access GO entries. The results of the KEGG pathway analysis were visualized on a bubble map.

### Molecular docking

Ligand–receptor interactions were simulated through molecular docking to predict the binding mode and energy. Initially, the sdf fragments of the three-dimensional structures of receptors were obtained using the PubChem database.^8^ Subsequently, Openbabel and Autodock were used to convert the identified fragments to the PDBQT format. Key proteins/ligands were identified from the Protein Data Bank (PDB) database,^9^ and the PyMol software was used for removal of water molecules, extraction of ligands, determination of docking boxes, and other necessary manipulations. AutoDock was used for hydrogenation and charge processing. The targets and drugs were prepared and molecular docking performed inside a grid box (40 Å × 40 Å × 40 Å). A Lamarckian genetic algorithm was used for protein-ligand docking with default settings, an exhaustiveness level of 8, and a maximum of 10 outputs. The outputs were downloaded in the PDBQT format. Finally, binding energy was evaluated through molecular docking, with lower values indicating more stable binding between the ligand and receptor.

### Molecular dynamics simulation

Molecular dynamics simulations of the ligand-receptor complexes were conducted using the GROMACS.^10^ The protein topology files were generated utilizing the AMBER99SB-ILDN force field, while the ligand topology files were created using the ACPYPE script under the AMBER force field. Then, the simulations were carried out in a triclinic box filled with TIP3 water molecules. Following system neutralization with NaCl counter ions, energy minimization was performed for 1,000 steps. Subsequently, the system was equilibrated through NVT and NPT ensembles for 100 ps each. Finally, molecular dynamics simulations were executed for 100 ns per system under periodic boundary conditions at a temperature of 310 K and 1.0 bar pressure.

## Experimental verification

### Materials

Fluorouracil (5-FU, HY-90006), Quercetin (HY-18085), Kaempferol (HY-14590), Beta-Sitosterol (HY-N0171A), and Stigmasterol (HY-N0131) were all obtained from the MedChemExpress. IL-6 (BE6304H1), AKT1 (BE03171H1), CASP3 (BE04705H1), CCND1 (BE5634H1), CTNNB1 (BE5900H1) and EGFR (BE04853H1) were all obtained from MCE. IL-1β was all obtained from the Yuli Biotechnology Co. Ltd., Shanghai.

### Cell culture

HACAT cells (Human immortal keratinocyte line) were obtained from the Asia-Vector Biotechnology (Shanghai) Co.Ltd. HACAT cells were cultured in DMEM (dulbecco’s modified eagle medium) with 10% fetal bovine serum (FBS).

### Cell counting Kit-8 assay

Cells in the logarithmic phase of growth (2000 cells/well) were cultured on 96-well plates filled with 150 μL complete medium for 1 day. After different treatments, CCK-8 solution (10 μL, Sigma, Germany) was added and cultured for 1 h at 37°C. After that, the optical density of each well at 450 nm was detected.

### Protein expression

IL-6 and IL-1 β in cell culture supernatants were confirmed by ELISA using the ELISA Kit (Asia-Vector Biotechnology Co. Ltd., Shanghai) as per the manufacturer’s instructions. AKT1, CASP3, CCND1, CTNNB1, and EGFR in cells were confirmed by ELISA using the ELISA Kit (Asia-Vector Biotechnology Co. Ltd., Shanghai) as per the manufacturer’s instructions.

### Statistical analysis

All experiments were independently performed at least three times. Data were represented as mean ± standard deviation. Means between the two groups were compared using the one-way ANOVA, with Dunnett’s correction (GraphPad Software). Differences were considered significant at *p* < 0.05. Statistical tests are indicated in the figure legends: *p < 0.05; **p < 0.01; ***p < 0.001; ****p < 0.0001.

## Results

### Active components and targets of SPSCG

A comprehensive search in the TCMSP database revealed active components in safflower, phellodendron, scutellaria baicalensis, and gardenia, respectively. Notably, the identified active components primarily contained the quercetin, kaempferol, β-sitosterol, and stigmasterol (Table 1). Subsequently, 1,212 target genes of these components were identified. After gene normalization and weight removal using the UniProt database, 197 target genes were anchored. Table 2 shows the drug metabolism and molecular structure of active ingredients including quercetin, kaempferol, β-sitosterol, and stigmasterol.

**Table 1:** Relevant information about the active ingredients of safflower, phellodendron, scutellaria baicalensis, coptis, and gardenia.

Mol ID	Compound	Degree value	Traditional Chinese Medicine
MOL000098	Quercetin	488	Safflower, phellodendron, scutellaria baicalensis, and gardenia
MOL000358	β-sitosterol	100	Safflower, phellodendron, scutellaria baicalensis, and gardenia
MOL000449	Stigmasterol	100	Safflower, phellodendron, scutellaria baicalensis, and gardenia
MOL000422	Kaempferol	92	Safflower and gardenia

**Table 2:** Drug metabolism of active ingredients.

Liver drug enzyme	β-sitosterol	Stigmasterol	Quercetin	Kaempferol
CYP1A2 inhibitor	0.044 (−--)	0.042 (−--)	0.942 (+++)	0.972 (+++)
CYP1A2 substrate	0.491 (−)	0.622 (+)	0.115 (−-)	0.11 (−-)
CYP2C19 inhibitor	0.074 (−--)	0.075 (−--)	0.053 (−--)	0.181 (−-)
CYP2C19 substrate	0.958 (+++)	0.962 (+++)	0.041 (−--)	0.046 (−--)
CYP2C9 inhibitor	0.096 (−--)	0.111 (−-)	0.598 (+)	0.653 (+)
CYP2C9 substrate	0.314 (−)	0.231 (−-)	0.643 (+)	0.867 (++)
CYP2D6 inhibitor	0.005 (−--)	0.027 (−--)	0.411 (−)	0.722 (++)
CYP2D6 substrate	0.409 (−)	0.737 (++)	0.205 (−-)	0.283 (−-)
CYP3A4 inhibitor	0.202 (−-)	0.342 (−)	0.348 (−)	0.697 (+)
CYP3A4 substrate	0.784 (++)	0.882 (++)	0.046 (−--)	0.08 (−--)
Molecular structure	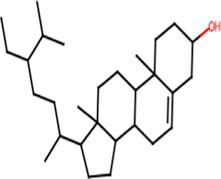	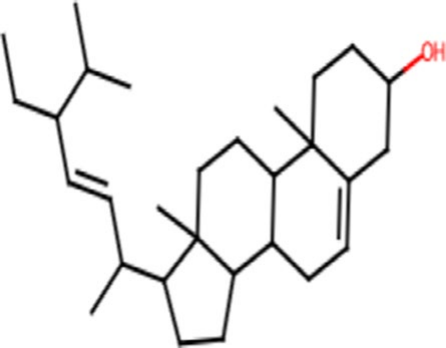	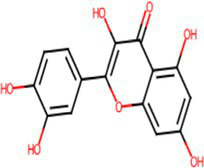	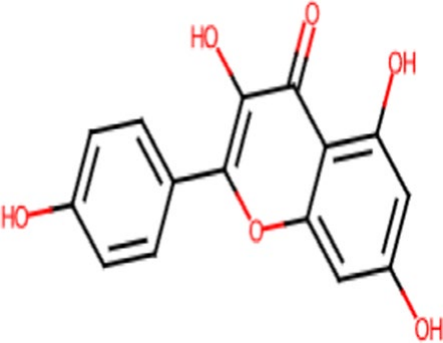

### Identification of HFS-related genes

**Figure 1:**
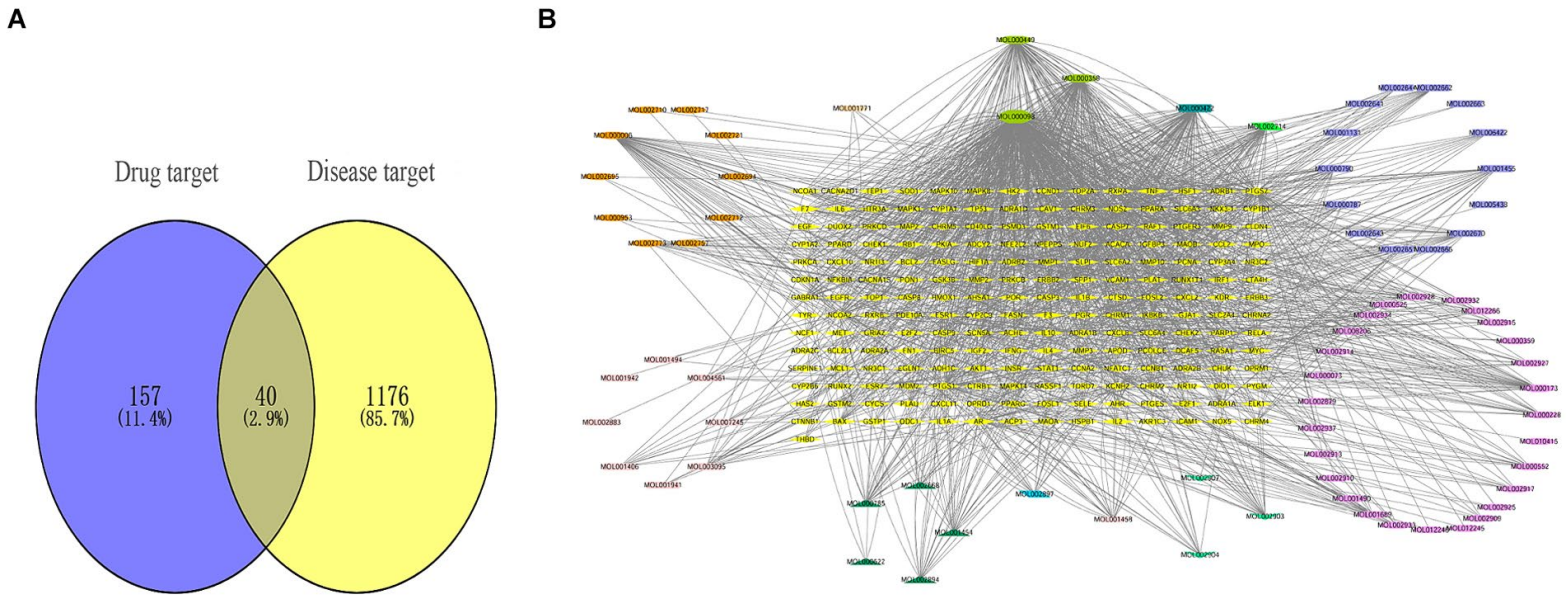
Composite target relationship between safflower, phellodendron, scutellaria baicalensis, coptis, gardenia and hand-foot syndrome. **(A)** Wayne diagram of target for intersection of safflower, phellodendron, scutellaria baicalensis, coptis, and gardenia with hand-foot syndrome. **(B)** Safflower, phellodendron, scutellaria baicalensis, coptis, and gardenia-active ingredient-the target network plot.

Firstly, we obtained a total of 179 drug targets using the database. Then, we identified 1,216 targets for HFS using Gene Cards, OMIM, TTD, Pharm GKB, and Drug Bank databases with the keyword “hand-foot syndrome.” The Venny 2.1.0 tool was used to intersect the 197 targets of SPSCG and 1,216 HFS-related targets, resulting in the identification of 40 overlapping targets (Figure 1A).

### Construction of the SPSCG-active component-target network

The SPSCG-active component–target network was constructed using the Cytoscape (version 3.7.2) software (Figure 1B). The network comprised 95 active components of SPSCG, which had a total of 40 common targets. In particular, 16, 32, 24, 11, and 12 active components belonged to safflower, scutellaria baicalensis, phellodendron, coptis chinensis, and gardenia, respectively. Based on the degree value, the top four active components in the network were quercetin, kaempferol, β-sitosterol, and stigmasterol. Kaempferol was identified as a common component of two herbs (Table 1), whereas quercetin, β-sitosterol, and stigmasterol were identified as common components of four herbs (Table 1). These four active components (Table 1) may contribute to the anti-inflammatory effects of SPSCG in treating HFS.

### Construction of the PPI network

**Figure 2:**
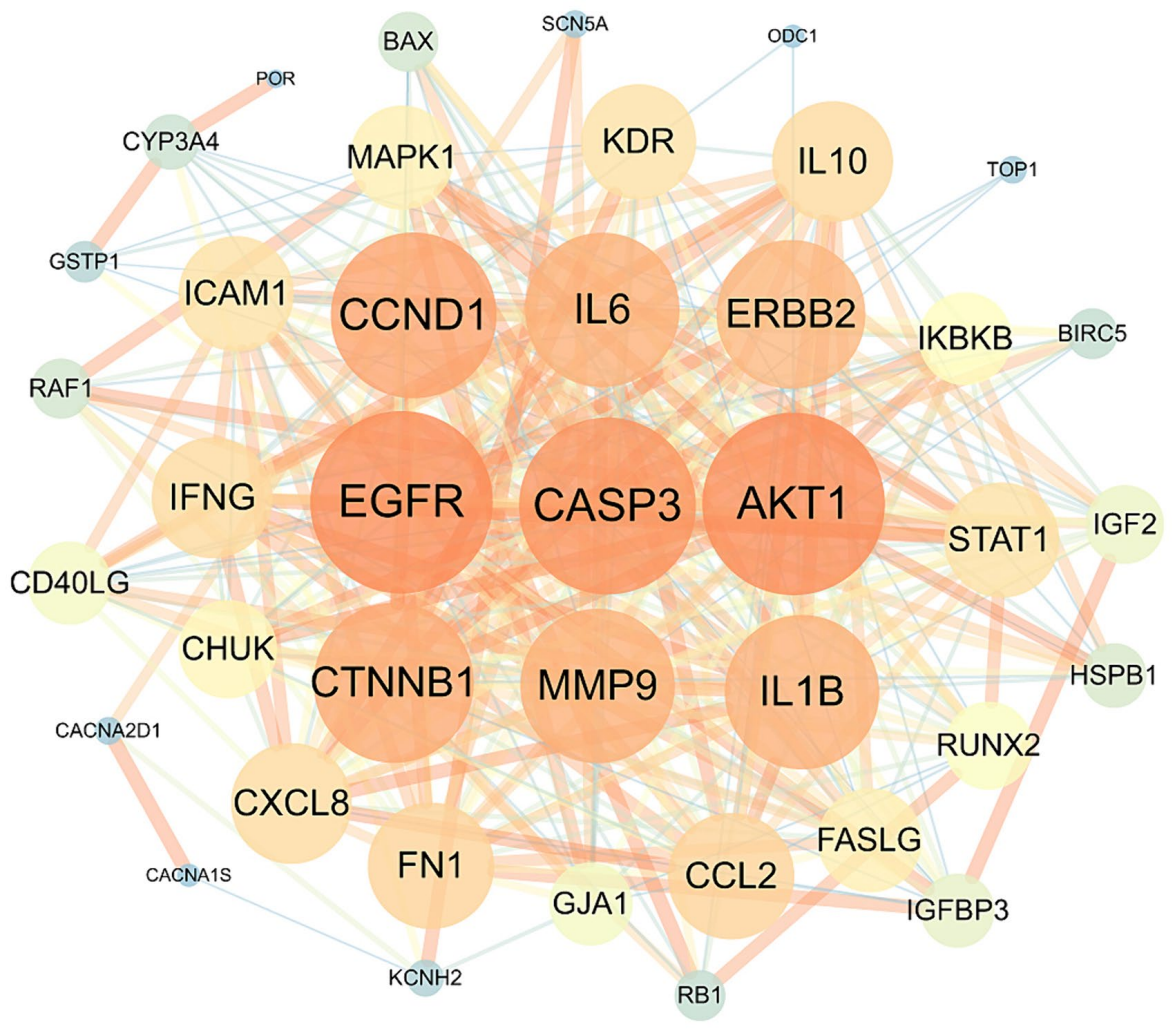
The protein–protein interaction network of safflower, phellodendron, scutellaria baicalensis, coptis, and gardenia with the target proteins.

The overlapping targets were submitted to the STRING (version 11.0) database, with the organism species specified as “*Homo sapiens*” and the minimum interaction threshold set to “medium confidence” (>0.4). Subsequently, the TSV file was downloaded and used to generate the PPI network of the overlapping targets using Cytoscape. In the resulting network, nodes with larger areas and font sizes were shown in dark red, indicating higher degree values and importance of the corresponding targets within the network. Based on the degree value, interleukin (IL)-6, CCND1, EGFR, CASP3, AKT1, and CTNNB1 were identified as the top six core targets (Figure 2). These target genes may play a crucial role in treating HFS.

### Enrichment analysis of SPSCG-HFS-related targets

**Figure 3:**
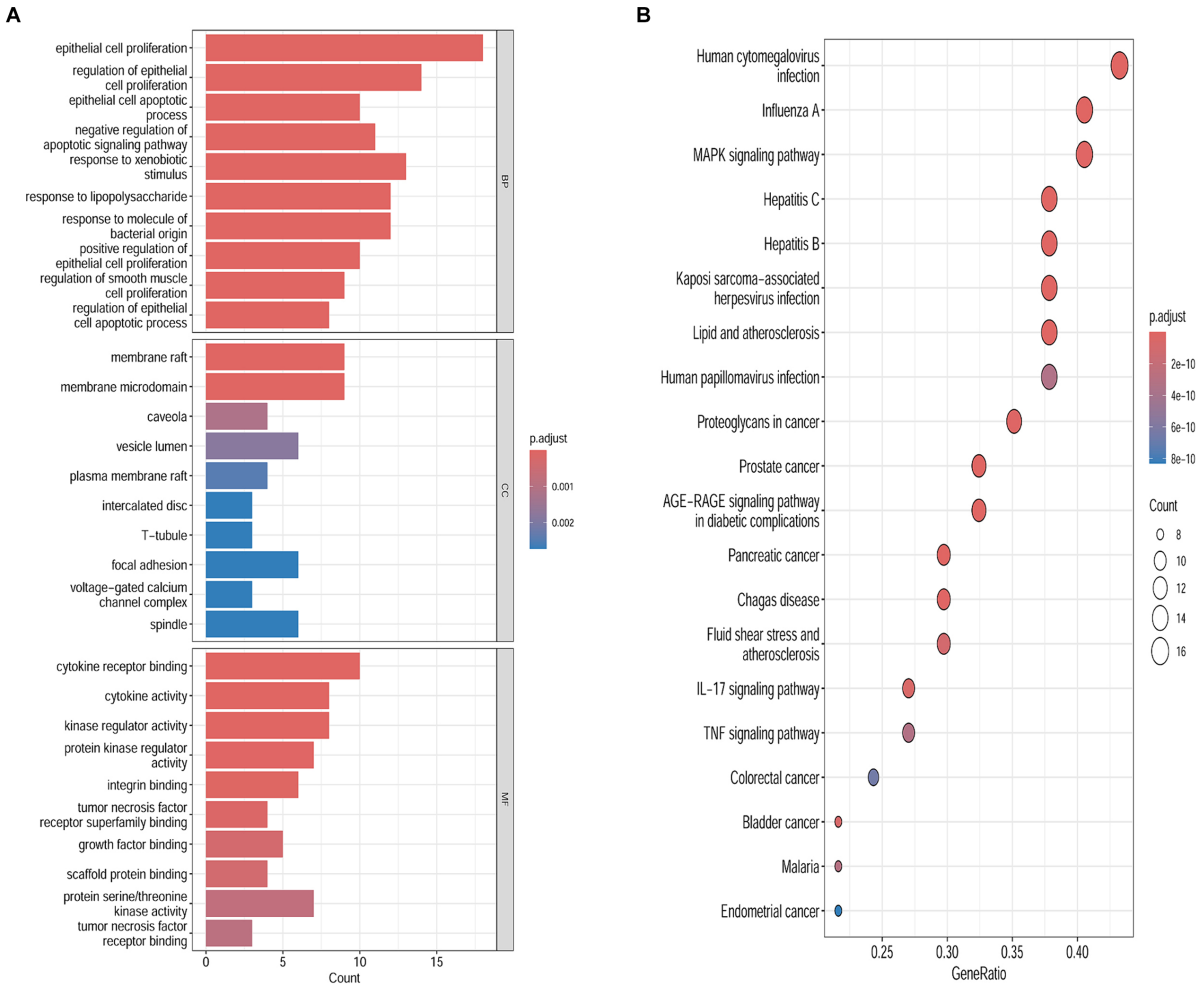
Biological functions and the pathways enrichment analyses. **(A)** Genomes pathway analysis of the intersection targets of safflower, phellodendron, scutellaria baicalensis, coptis, and gardenia and hand-foot syndrome (BP/CC/MF). **(B)** Kyoto Encyclopedia of Genes analysis of the intersection targets of safflower, phellodendron, scutellaria baicalensis, coptis, and gardenia and hand-foot syndrome.

To examine the correlation between target genes and the biological phenotypes of HFS, the 40 common targets were subjected to GO and KEGG analysis using the Metascape database. The results showed that the target genes were significantly enriched in 1226 GO terms (1,127 BPs, 42 CCs, and 57 MFs) and 149 KEGG pathways (*p* < 0.01). A Cytoscape 3.9.0 online platform was used to visualize the top 10 GO terms on a three-in-one bar chart and the top 20 KEGG pathways on a bubble chart. Based on GO analysis, the target genes were primarily enriched in BPs, such as epithelial cell proliferation and positive regulation of epithelial cell proliferation. CCs, such as cell–cell contact zone and focal adhesion; and MFs such as cytokine receptor binding, growth factor binding, and scaffold protein binding (Figure 3A). KEGG analysis showed that the target genes were primarily enriched in the MAPK signaling pathway and other related pathways (Figure 3B).

### Molecular docking

**Figure 4:**
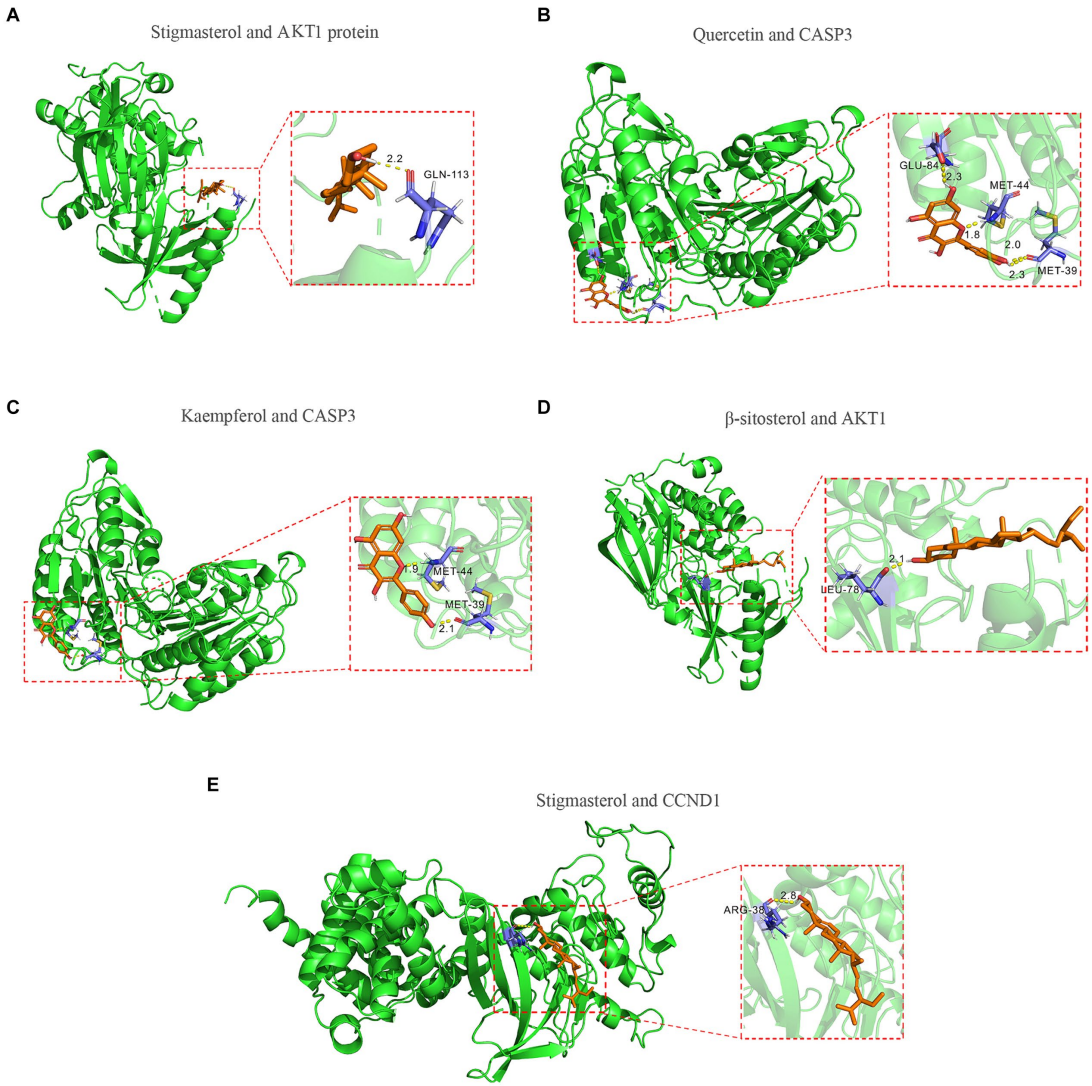
Molecular docking of core components of safflower, phellodendron, scutellaria baicalensis, coptis, and gardenia to key targets figure. **(A)** Stigmasterol and AKT1 protein. **(B)** Quercetin and CASP3 protein. **(C)** Kaempferol and CASP3 protein. **(D)** β-sitosterol and AKT1 protein. **(E)** Stigmasterol and CCND1.

To further validate the predictive capability of bioinformatics, molecular docking was employed to investigate the potential of SPSCG in treating HFS. Following selection via CytoNCA analysis, the most critical HFS targets (AKT1, CASP3, and CCND1) were subjected to molecular docking with SPSCG (Figures 4A–E). As shown in Figures 4B,C, there were four and two hydrogen bonds in Quercetin-CASP3 and Kaempferol-CASP3, respectively. In Figures 4A,D,E, each had one hydrogen bond in Quercetin-CASP3, β-sitosterol-AKT1, and Stigmasterol-CCND1. Table 3 shows the hydrogen bond distance, interacting residues and unit of docking score. As depicted in Table 3, we checked the hydrogen bond distances in docked complexes. The hydrogen bond distances of Stigmasterol-CCND1, Quercetin-CASP3, Kaempferol-CASP3, β-sitosterol-AKT1, and Stigmasterol-CCND1 were 2.8, (2.3; 1.8; 2.0; 2.3), (1.9; 2.1), 2.1, and 2.8 angstroms, respectively. The maximum binding free energies for AKT1, CASP3, CCND1, CTNN1, and EGFR with the quercetin, β-sitosterol, stigmasterol, and kaempferol were − 4.93, −5.2, −4.09, −3.03, and − 3.65, respectively (Table 3). The docking binding free energies of the aforementioned molecules were all less than −5, indicating the strong binding affinity of SPSCG to the core targets of HFS. The cartoon representation highlights the specific locations of the ligand-protein binding residues (Figures 4A–E).

**Table 3:** The docking of safflower, phellodendron, scutellaria baicalensis, coptis, and gardenia core components with key target molecules.

Compound	Gene	binding energy (kcal/mol)	interacting residues	hydrogen bond distance (angstroms)
Quercetin	CASP3	−5.2	None	None
Stigmasterol	AKT1	−4.93	GLN-113	2.2
β-sitosterol	CASP3	−4.78	None	None
Quercetin	CASP3	−4.6	GLU-84; MET-44; MET-39	2.3; 1.8; 2.0,2.3
Kaempferol	CASP3	−4.41	MET-44; MET-39	1.9; 2.1
β-sitosterol	AKT1	−4.19	LEU-78	2.1
β-sitosterol	CCND1	−4.14	None	None
Stigmasterol	CCND1	−4.09	ARG-38	2.8
Stigmasterol	EGFR	−3.65	None	None
β-sitosterol	CTNNB1	−3.56	GLN-482	2.3
Kaempferol	AKT1	−3.48	LYS-268	2.1
β-sitosterol	EGFR	−3.4	None	None
Quercetin	AKT1	−3.35	THR-34; ASP-32; SER-56; GLN-59; ASN-53	2.5; 2.2; 2.4; 2.9; 2.3
Kaempferol	CCND1	−3.24	None	None
Kaempferol	EGFR	−3.13	LEU-201	2
Stigmasterol	CTNNB1	−3.03	None	None
Kaempferol	CTNNB1	−2.91	THR-547; ARG-549	2.5; 2.6
Quercetin	EGFR	−2.91	GLU-143; TYR-173	3.0,2.5; 2.4
Quercetin	CCND1	−2.53	LYS-88; GLN-168	2.7; 2.2
Quercetin	CTNNB1	−2.35	None	None

### Molecular dynamics simulation

**Figure 5:**
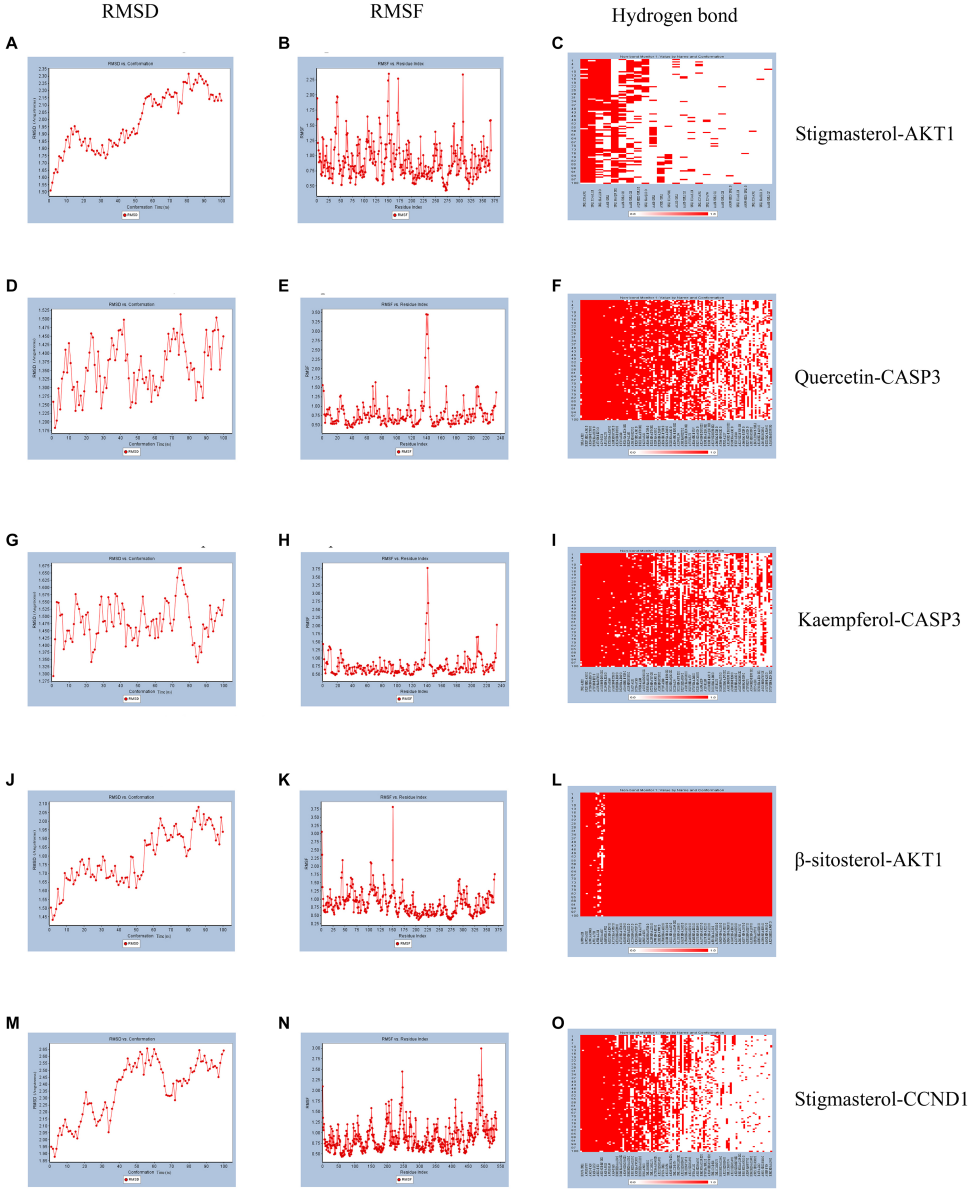
Molecular dynamics simulation. **(A)** The RMSD of Stigmasterol-AKT1. **(B)** The RMSF of Stigmasterol-AKT1. **(C)** The hydrogen bonds of Stigmasterol-AKT1. **(D)** The RMSD of Quercetin-CASP3. **(E)** The RMSF of Quercetin-CASP3. **(F)** The hydrogen bonds of Quercetin-CASP3. **(G)** The RMSD of Kaempferol-CASP3. **(H)** The RMSF of Kaempferol-CASP3. **(I)** The hydrogen bonds of Kaempferol-CASP3. **(J)** The RMSD of β-sitosterol-AKT1. **(K)** The RMSF of β-sitosterol-AKT1. **(L)** The hydrogen bonds of β-sitosterol-AKT1. **(M)** The RMSD of Stigmasterol-CCND1. **(N)** The RMSF of Stigmasterol-CCND1. **(O)** The hydrogen bonds of Stigmasterol-CCND1.

According to molecular docking results, the binding force between the compound and the protein increases as the binding free energies decrease. We used molecular dynamics simulations to validate the binding mode between Quercetin and CASP3, Kaempferol and CASP3, Stigmasterol and AKT1, β-sitosterol and AKT1, Stigmasterol and CCND1. And the obtained RMSD, RMSF, and hydrogen bond were used to evaluate their relative stability. The trend of RMSD of the protein and ligand complexes is a crucial indicator of whether the simulation has reached stability. As depicted in Figures 5A,D,G,J,M, the conformation reached a relatively stable state at approximately 20, 5, 3, 15, and 10 ns respectively, and fluctuated around 1.8–2.3, 1.25–1.5, 1.425–1.575, 1.65–2.05, and 2.05–2.65 angstroms, respectively. Complexes with small fluctuations were relatively stable, and those with large fluctuations were relatively unstable. RMSF values quantify a protein’s structural stability, atomic mobility, and residual flexibility upon collision. As depicted in Figures 5B,E,H,K,N, each system also exhibited different RMSF fluctuation trends and flexible regions. Hydrogen bonds indicate the binding strength between the ligand and the protein. The docked complex had a stable pattern of hydrogen bonds (Figures 5C,F,I,L,O). Complexes with hydrogen bonds are relatively stable. The molecular dynamics simulations results of hydrogen bonds were correlated with the dokcing results. Thus, the molecular dynamics simulations demonstrate that Stigmasterol-CCND1, Quercetin-CASP3, Kaempferol-CASP3, β-sitosterol-AKT1, and Stigmasterol-CCND1 had relatively good binding ability.

### Cell survival rate and cytokine expression level

**Figure 6:**
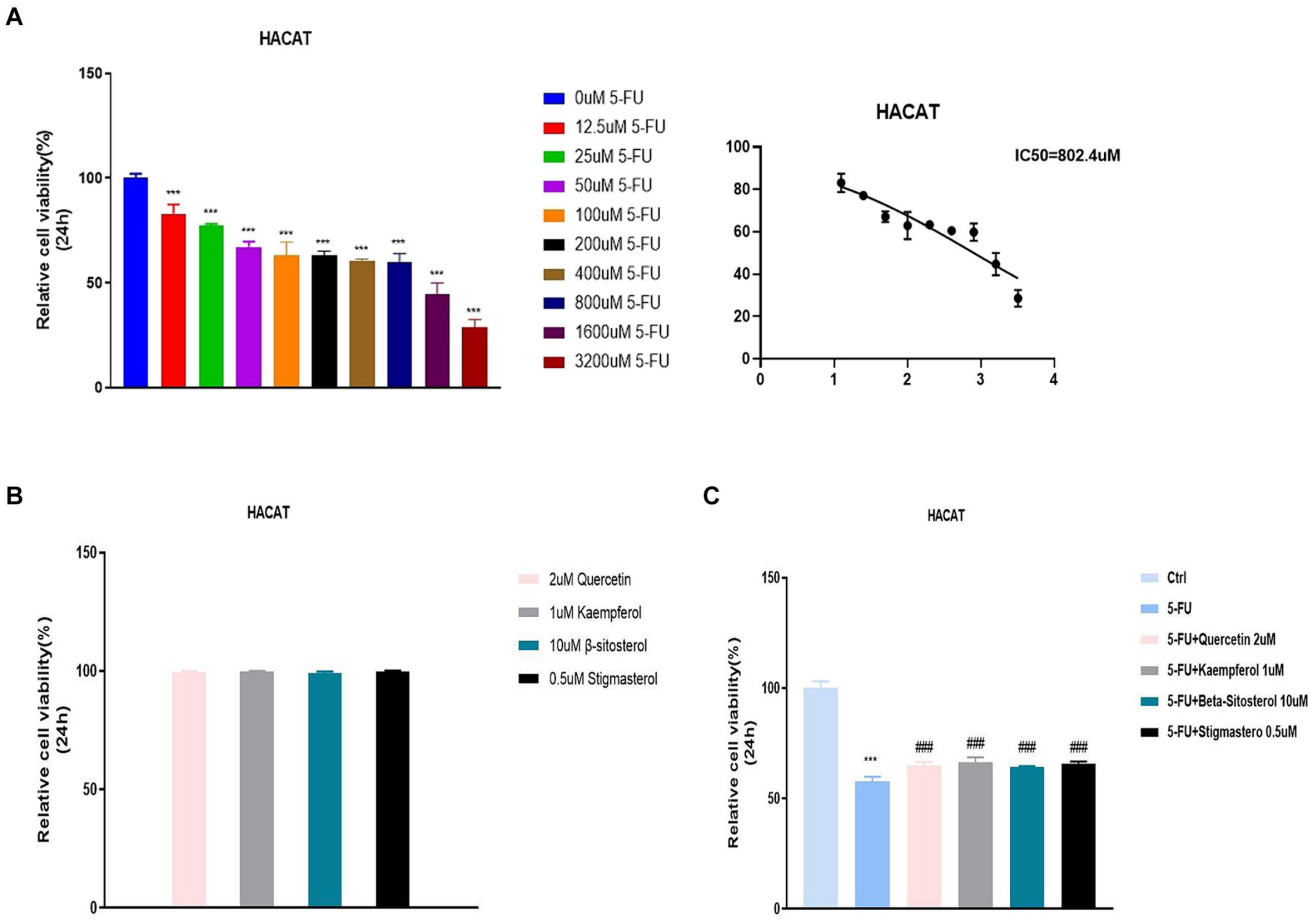
Relative cell viability in different concentration groups in HACAT cells. **(A)** After the treatment with different concentrations of 5-FU for 24 h, the relative cell viability was tested by CCK 8 (IC_50_). The IC_50_ of 5-FU was the 802.4uM, which was selected for the downstream experiments. **(B)** After the treatment with 2uM Quercetin, 1uM Kaempferol, 10uMβ-sitosterol and 0.5uM Sigmasterol for 24 h, the relative cell viability was tested by CCK 8 (IC_50_). **(C)** After the treatment with 5-FU, 5-FU + 2uM Quercetin, 5-FU + 1uM Kaempferol, 5-FU + 10uM β-sitosterol and 5-FU + 0.5uM Sigmasterol for 24 h, the relative cell viability was tested by CCK 8 (IC_50_). Data represent mean ± SD. *** vs. Ctrl *p* < 0.001; ^###^ vs. 5-FU *p* < 0.001 (one-way ANOVA, with Dunnett’s correction). Significance level, α = 0.05.

The previous data were obtained from public databases. Next, HACAT cells were cultured *in vitro* and cytotoxicity was tested with fluorouracil. The optimal dose of 5-FU at 50% cell survival was 802.4uM (Figure 6A). When quercetin (2 uM), kaempferol (1 uM), β-sitosterol (10 uM), and stigmasterol (0.5 uM) were added, the cell survival rate was 100%, indicating that the TCM had no inhibitory effect on cell growth (Figure 6B). When added with quercetin, kaempferol, β-sitosterol, and stigmasterol, the survival rate of cells was higher than that of cells added with 5-FU alone (Figure 6C; all *p* < 0.0001). This indicated that the cell growth-promoting effect of TCM could antagonize the inhibitory effect of 5-FU.

**Figure 7:**
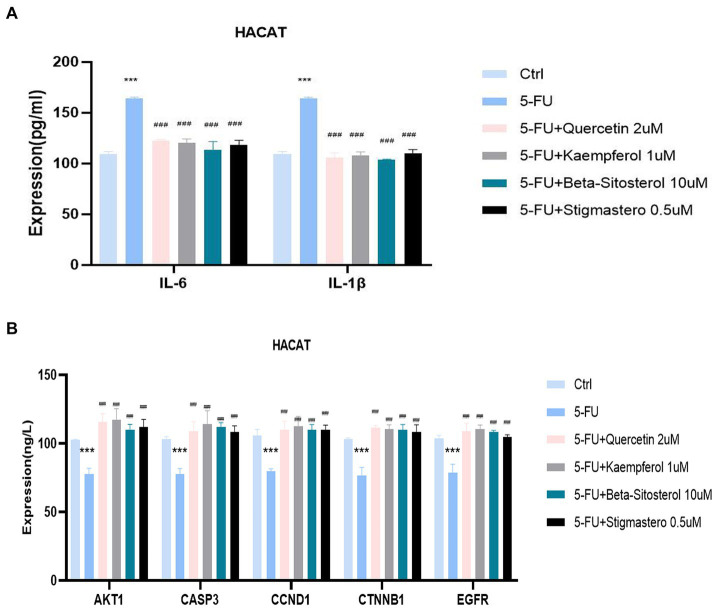
Comparison of the expression of different cytokines and targets in different groups including control (Ctrl), 5-FU, 5-FU + 2uM Quercetin, 5-FU + 1uM Kaempferol, 5-FU + 10uM β-sitosterol, and 5-FU + 0.5uM Sigmasterol. **(A)** Comparison of the expression of IL-6 and IL-1β in different groups. The test sample was the supernatant taken after 24-h culture. **(B)** Comparison of the expression of different AKT1, CASP3, CCND1, CTNNB1, and EGFR in different groups. The test sample was the cells taken after 24-h culture. Data represent mean ± SD. *** vs. Ctrl *p* < 0.001; ^###^ vs. 5-FU *p* < 0.001 (one-way ANOVA, with Dunnett’s correction). Significance level, *α* = 0.05.

Figure 7A shows that IL-6 and IL-1 β expression were significantly increased in the cells added with 5-FU, when compared with the normal cell group. When added with quercetin, kaempferol, β-sitosterol, and stigmasterol, the IL-6 and IL-1 β levels were lower than that of cells added with 5-FU alone (Figure 7A; all *p* < 0.0001), and the expression of AKT1, CASP3, CCND1, CTNNB1,and EGFR were higher than that of cells added with 5-FU alone (Figure 7B; all *p* < 0.0001).

## Discussion

Network pharmacology involves the identification of drug targets and disease-related genes to assess the therapeutic efficacy. This approach offers the advantages of an increased success rate and the decreased drug development expenses (23). In this study, data from various platforms including network pharmacology were integrated and intersected to investigate the key component of SPSCG in HFS (24). The mechanism of SPSCG in the treatment of HFS was predicted, which warrants subsequent experimental validation.

The precise etiology of HFS remains uncertain, and the mechanisms through which anti-cancer drugs induce HFS may vary. HFS is characterized by varying degrees of necrosis in cells and inflammation at the junction of the epidermis and dermis (4). Additionally, some patients could develop exocrine gland squamous epithelial granuloma or neutrophil-infiltrating hidradenitis (25). Microscopic examination revealed vacuolar degeneration, infiltration by skin basal keratinocytes and perivascular lymphocytes, vasodilation, and edema (26), resembling an inflammatory response. Owing to the abundant capillary networks and higher density of secretory glands in the palms and soles, these sites might accumulate excess chemotherapy drugs (27). Consequently, inflammatory responses are more pronounced in the palms and soles. This phenomenon suggests that HFS can be alleviated and managed by suppressing inflammatory responses. Given that SPSCG is widely used in TCM, this study may present an innovative approach to investigating the mechanism of SPSCG in HFS treatment.

The primary active components of SPSCG contains the quercetin, kaempferol, β-sitosterol, and stigmasterol. Studies have shown that quercetin has antioxidative properties, protects the vascular endothelium, and inhibits inflammatory responses (28). Furthermore, it can decrease the expression of PIK3R1 and EGFR (29), contributing to the repair of skin damage through the PI3K-AKT and EGFR/MAPK pathways. Kaempferol is commonly found in various edible plants and is used to formulate natural medications in TCM (30). It has multiple pharmacological activities, including antioxidative and anti-inflammatory activities (30). Additionally, it could inhibit macrophage inflammation (31) and exert cytoprotective and anti-apoptotic effects (32). β-sitosterol plays an essential role in stabilizing the phospholipid bilayer of the cell membrane (33). It exhibits lipid-lowering, anti-inflammatory, and antioxidative activities (34). It has been shown to mitigate inflammatory response through inhibition of the ERK/p38 and NF-κB pathways induced by LPS in BV2 cells (35). Stigmasterol holds significant therapeutic potential owing to its diverse pharmacological effects, including anti-inflammatory (36) and antioxidative effects (37). It can increase the expression of anti-inflammatory cytokines, particularly IL-10. Conversely, it can notably increase the expression of pro-inflammatory mediators, including iNOS, TNF-α, IL-1β, IL-6, and COX-2. These findings suggest that stigmasterol can effectively alleviate inflammation by suppressing the expression of pro-inflammatory cytokines (38). Therefore, SPSCG’s core active ingredients combat inflammation and aid skin repair via antioxidant, anti-inflammatory, and cell-protective mechanisms.

In this study, the primary targets of SPSCG for the treatment of HFS were identified as CASP3, EGFR, AKT1, CCND1, and CTNNB1. Caspase 3 played an important role in regulating skin damage, as it was implicated in apoptotic pathways characterized by genomic DNA cleavage, cell membrane phosphatidylserine exposure, and activation by inflammatory cells (39). Inhibition of the CASP3 sustained the inactive phosphorylated state of the FoxO3a, effectively preserving endothelial cell integrity (40). EGFR is a crucial signaling molecule involved in maintaining the structure and functions of the skin and regulating inflammatory and immune responses in the skin (41). Inhibition of EGFR in cuticular cells could aggravate skin inflammation (42). Moreover, EGFR was downregulated at both mRNA and protein levels in dermatitis (43). In this study, stigmasterol was found to exhibit the highest binding affinity to EGFR, suggesting that EGFR might be a promising target for stigmasterol-based treatment in HFS. AKT1 is involved in regulating various biological processes, such as cell proliferation and angiogenesis. Activation of AKT signaling was facilitated by phosphatidylinositol 3-phosphoinositide 3-kinase (PI3K). AKT1 served as a crucial downstream target of the PI3K signaling pathway, contributing to cellular homeostasis during the differentiation of peripheral blood B and T cells (44). The pathological characteristics of HFS include skin damage and inflammation. Hence, alleviating these symptoms represents an effective therapeutic strategy for HFS. EGFR and AKT1 can counteract inflammation. Nonsteroidal Antiinflammatory Drugs can regulate cellular glycosaminoglycan synthesis by modulating the EGFR and PI3K signaling pathways (45). In this study, β-sitosterol and stigmasterol were found to have significant binding affinity to EGFR and AKT1. We speculate that EGFR/AKT signaling is one of the important pathways that SPSCG functions in HFS treatment. Cyclin D1 exhibits frequent abnormalities in various human cancers (46) and plays a crucial role in facilitating the transition from the G1 to the S phase of the cell cycle in numerous cell types (47). The proximal selective polyadenylation sites of CCND1 have been identified as accelerators of the cell cycle and promoters of cell proliferation (48). In this study, β-sitosterol and CCND1 had the highest binding scores, suggesting that β-sitosterol targets CCND1 to regulate the cell cycle for the HFS treatment. The CTNNB1 gene encodes β-catenin, which serves as a crucial component of the Wnt/β-catenin signaling pathway and plays an essential role in cadherin-mediated intercellular adhesion. Numerous studies have shown that downregulation of β-catenin leads to inhibition of cell growth and induction of apoptosis in diverse cell types (49). In the present study, β-sitosterol exhibited the most notable binding affinity to CTNNB1. This finding suggests the involvement of CTNNB1 in cellular regeneration following skin injury, thereby establishing it as a crucial target of β-sitosterol in the management of HFS.

We found that the treatment of HFS with SPSCG involves several interconnected pathways, such as the MAPK, TNF, and IL-17 signaling pathways by GO and KEGG analysis. HFS is a prevalent adverse event associated with various chemotherapeutic drugs and tumor-targeted therapies (2). The pathogenesis of HFS includes the formation of skin keratinocytes, increased levels of thymidine phosphatase, required for the degradation of drugs such as 5-fluorouracil, and result in augmented breakdown and accumulation of cytotoxic metabolites in the skin (50). Furthermore, the vulnerability of minute capillaries in the skin causes ruptures in the palms of the hands and the soles of the feet owing to the pressure exerted during walking or use, thereby releasing cytotoxic agents and inducing inflammatory responses (51). Notably, drugs targeting atopic dermatitis could decrease p38 MAPK levels and inhibit the activation of the NF-κB signaling pathway (52). Therefore, MAPK inhibitors might serve as effective therapeutic agents for skin inflammation caused by allergic reactions (53). Suppression of MAPK signaling and modulation of intracellular signaling pathways associated with atopic inflammation has been shown to alleviate the symptoms of atopic dermatitis (54) (55). Consequently, activating the EGFR/AKT signaling pathway while inhibiting MAPK activity can effectively mitigate inflammation and enhance the integrity of the skin barrier, representing a promising strategy for the treatment of HFS. During inflammatory reactions, TNF-α could trigger non-specific immune responses by activating macrophages and promoting the release of additional inflammatory cytokines (56). TNF-α is released during the early stages of both acute and chronic inflammatory conditions, such as septic shock, rheumatoid arthritis, and allergic reactions. The biosynthesis of TNF-α is regulated through intricate and diverse mechanisms, primarily involving gene transcription, mRNA degradation, and intracellular protein signaling (57). SPSCG may inhibit the secretion of TNF-α. However, the underlying mechanisms warrant further investigation.

Our experiment confirmed that the quercetin, kaempferol, β-sitosterol and stigmasterol have a promoting effect on epithelial cell growth at appropriate concentrations, which could antagonize the cytotoxic effects caused by 5-FU. The quercetin, kaempferol, β-sitosterol, and stigmasterol downregulated the inflammatory factors and upregulated cell proliferation-related factors caused by 5-FU. Therefore, the quercetin, kaempferol, β-sitosterol, and stigmasterol may treat HFS for their anti-inflammatory, antioxidant, and cell damage repair effects. However, we did not establish an animal model to validate the repair effect of the core drugs on HFS.

There are several limitations to this study. The network pharmacology approach improves the investigation of natural plant products used in TCM but faces challenges owing to incomplete data and evaluation criteria. In the future study, we want to verify it with clinical studies. Secondly, *in vivo* experiments should be integrated to validate the targets and pathways of natural products to expand the clinical application of TCM.

## Conclusion

In summary, quercetin, kaempferol, β-sitosterol, and stigmasterol are the active components of SPSCG, which can target AKT1, CASP3, CCND1, CTNNB1, and EGFR to exert anti-inflammatory and antioxidative effects for treating HFS. However, further investigation is required to validate these findings and elucidate the mechanisms of the abovementioned active components in treating HPS.

## Data Availability

The original contributions presented in the study are included in the article/supplementary material, further inquiries can be directed to the corresponding author/s.

## Footnotes

^1^ https://admetmesh.scbdd.com/

^2^ https://www.uniprot.org/

^3^ https://disgenet.org

^4^ http://www.omim.org

^5^ https://www.genecards.org

^6^ https://string-db.org/

^7^ https://metascape.org/

^8^ https://pubchem.ncbi.nlm.nih.gov/

^9^ https://www.rcsb.org/

^10^version 2021.2, https://www.gromacs.org/
